# Clinical Characteristics at the Diagnosis of New Primary Melanoma in Italy: A Multicenter Retrospective Study Before and After the COVID-19 Pandemic

**DOI:** 10.3390/jcm15072715

**Published:** 2026-04-03

**Authors:** Elisabetta Pennacchioli, Luca Nespoli, Dario Piazzalunga, Virginia Caliendo, Piero Rossi, Marco Clementi, Matteo Mascherini, Ferdinando Cananzi, Salvatore Asero, Corrado Caracò, Paolo Carcoforo, Paolo Del Fiore, Sara Coppola, Martina Pellegrini, Chiara Trevisiol, Franco Picciotto, Maria Gabriella Valente, Cosimo Di Raimondo, Irene Tucceri Cimini, Franco De Cian, Samà Laura, Francesco Cavallin, Alessandra Buja, Pietro Gallina, Marco Rastrelli

**Affiliations:** 1 Soft Tissue Sarcomas and Rare Tumors, European Institute of Oncology, IRCCS, 20141 Milan, Italy; elisabetta.pennacchioli@ieo.it (E.P.);; 2 General and Emergency Surgery, School of Medicine and Surgery, Milano-Bicocca University, Fondazione IRCCS San Gerardo dei Tintori, Via Pergolesi 33, 20900 Monza, Italy; luca.nespoli@unimib.it (L.N.);; 3 Unit of Surgery, Papa Giovanni XXIII Hospital, 24100 Bergamo, Italy; 4 Department of Surgery, Dermatologic Surgery Section, Azienda Ospedaliera Universitaria (AOU) Città della Salute e della Scienza, 10126 Turin, Italyfranco.picciotto@gmail.com (F.P.); 5 Dipartimento Di Scienze Chirurgiche, Università di Roma Tor Vergata, Policlinico Tor Vergata Viale Oxford 81, 00133 Roma, Italycosimodiraimondo@gmail.com (C.D.R.); 6 General Surgical Unit, Department of Biotechnological and Applied Clinical Sciences, University of L’Aquila, 67100 L’Aquila, Italy; 7 Department of Surgical Sciences and Integrated Diagnostic DISC, University of Genoa, 16100 Genoa, Italy; 8 Department of Surgery, IRCCS Ospedale Policlinico San Martino, 16132 Genoa, Italy; 9 Sarcoma, Melanoma and Rare Tumors Surgery Unit, IRCCS Humanitas Research Hospital, 20089 Milan, Italy; 10 Soft Tissue U.O. Surgical Oncology-Soft Tissue Tumors, Dipartimento di Oncologia, Azienda Ospedaliera di Rilievo Nazionale e di Alta Specializzazione Garibaldi Catania, 95123 Catania, Italy; 11 Division of Surgery of Melanoma and Skin Cancer, Istituto Nazionale Tumori ‘Fondazione Pascale’ IRCCS, 80131 Naples, Italy; 12 Department of Medical Sciences, Section of General Surgery, University of Ferrara, 44121 Ferrara, Italy; 13 Soft-Tissue, Peritoneum and Melanoma Surgical Oncology Unit, Veneto Institute of Oncology IOV-IRCCS, 35128 Padua, Italymarco.rastrelli@unipd.it (M.R.); 14 Independent Statistician, 36020 Solagna, Italy; 15 Department of Cardiological, Thoracic and Vascular Sciences, and Public Health, University of Padua, Via Loredan, 18, 35127 Padua, Italy; 16 Directorate General, Veneto Institute of Oncology IOV-IRCCS, 35128 Padua, Italy; pietro.gallina@iov.veneto.it; 17 Department of Surgery, Oncology and Gastroenterology (DISCOG), University of Padua, 35122 Padua, Italy

**Keywords:** melanoma, COVID-19, delayed diagnosis, Breslow thickness, sentinel lymph node biopsy, Italian multicenter study, health care access

## Abstract

**Background/Objectives**: During the pandemic, access to healthcare was severely disrupted, inevitably affecting melanoma diagnosis. While this was clearly evident during the pandemic itself, it is less clear whether clinical presentation has returned to baseline levels in subsequent years. Our study aimed to compare the current clinical presentation of melanoma with that in the pre-pandemic setting. **Methods**: We conducted a retrospective multicenter study involving Italian melanoma referral centers within the SICO network. Patients with a newly diagnosed primary cutaneous melanoma were included in the study and were grouped into four time periods: pre-pandemic (March 2019 to February 2020); pandemic (March 2021 to February 2022); the first post-pandemic year (March 2022 to February 2023); and the second post-pandemic year (March 2023 to February 2024). Our focus was on clinically relevant features at diagnosis, including Breslow thickness, ulceration, stage and sentinel lymph node status. We evaluated differences across periods using regression models that accounted for the multicenter design. **Results**: A total of 4938 patients were included in the study. Compared with the pre-pandemic period, melanomas diagnosed during and after the pandemic were thicker, more frequently ulcerated and more likely to be in stages II–III. The rate of sentinel lymph node positivity also increased. Notably, these patterns did not normalize over time, remaining evident even in the second post-pandemic year. The results were consistent after adjusting for age and sex. **Conclusions**: In this large Italian study, melanoma continues to be diagnosed at a later stage than in the pre-pandemic period. This persistent shift may reflect a combination of delayed access to care and ongoing system-level constraints. These findings emphasize the importance of restoring timely access to dermatological evaluation and reinforcing early detection strategies.

## 1. Introduction

Cutaneous malignant melanoma is a significant public health problem with a rapidly increasing incidence worldwide, particularly in younger populations [1]. The increasing incidence of melanoma poses a significant and multifaceted burden on healthcare systems worldwide. In fact, the prognosis and treatment costs of newly diagnosed melanoma heavily rely on early diagnosis and prompt treatment.

There is evidence that lockdown restrictions due to the COVID-19 pandemic (such as elimination of routine medical examinations and severely restricted access to follow-up examinations) led to diagnostic delays that may be associated with higher incidence of advanced melanomas at diagnosis, characterized by increased Breslow thickness, greater ulceration, and more advanced stages than in the pre-pandemic period [2,3,4,5]. In particular, one study evaluated the total impact of delays to melanoma diagnosis during the lockdowns imposed due to the pandemic, which may affect clinical outcomes and incur direct and indirect costs across Europe. While healthcare services gradually returned to normality in 2021, in part thanks to the rollout of vaccines, there were no large-scale studies to determine whether the trend of late diagnoses persisted into the second year of the pandemic and beyond. A multicentric retrospective study, conducted in Italy by melanoma centers of the Italian Oncological Surgery Society Network (Società Italiana di The Surgical Oncology Society (SICO)) specifically addressed this issue, revealing that even in the second year, patients presented with more advanced melanoma characteristics at diagnosis [5]. This observation was consistent across various skin cancer centers in Italy, irrespective of organizational changes encountered during the pandemic, and may be associated with delays in accessing the clinical pathway rather than a slowdown in the diagnostic process.

This study aimed to evaluate the characteristics of Melanoma at the time of diagnosis following the pandemic and to ascertain whether the clinical features at diagnosis have reverted to pre-pandemic levels or whether, two years after presentation, the characteristics of this malignancy have undergone alterations.

## 2. Materials and Methods

### 2.1. Study Design

This is a retrospective, observational, multicenter study assessing the indirect impact of the COVID-19 pandemic on the presentation of melanoma patients at the first and second years after the pandemic. The study involved the melanoma centers of the Italian Oncological Surgery Society Network (Società Italiana di Chirurgia Oncologica—SICO).

### 2.2. Patients

The study included all patients diagnosed and/or treated for primary cutaneous melanoma at the participating centers during the time intervals of interest. The inclusion criteria were a histologically confirmed diagnosis of cutaneous melanoma, 18 years or older at diagnosis, and clinical stage I–II–III.

### 2.3. Study Periods

**Figure 1:**
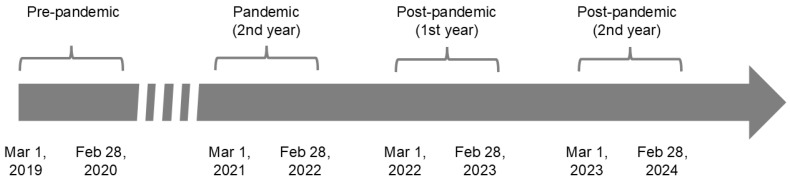
Timeline representing the study intervals: pre-pandemic (1 March 2019–28 February 2020), second year of the pandemic (1 March 2021–28 February 2022), first post-pandemic year (1 March 2022–28 February 2023), and second post-pandemic year (1 March 2023–28 February 2024).

The pre-pandemic period included all eligible patients from 1 March 2019 to 28 February 2020. The pandemic period under evaluation (second year) included all eligible patients from 1 March 2021 to 28 February 2022. The first post-pandemic period (first year) included all eligible patients from 1 March 2022 to 28 February 2023. The second post-pandemic period (second year) included all eligible patients from 1 March 2023 to 28 February 2024 (Figure 1). The analysis did not include data from the first year of the pandemic (from 1 March 2020 to 28 February 2021) because the study focused on the “mid-term” effects of the pandemic on melanoma diagnosis and treatment. This time interval included the first two waves of the pandemic when lockdown, strong limitations of mobility and unavailability of many health services occurred.

### 2.4. Outcome Measures

Clinically relevant tumor characteristics at the diagnosis included Breslow thickness (mm), tumor stage (TNM-AJCC stage; 8th edition), mitosis (units/mm^2^) and ulceration (yes/no). Indicators of disease progression at diagnosis included (i) the proportion of patients who underwent sentinel lymph node biopsy (SLNB), and (ii) the proportion of patients with positive nodes. All data were retrieved from the hospital charts and collected in an anonymized database for the analysis.

### 2.5. Statistical Analysis

The numerical variables were summarized as mean and standard deviation (SD), and the categorical variables as count and percentage. The indirect impact of the pandemic was evaluated using linear regression models and logistic regression models including the pre-pandemic period (as baseline), the pandemic period (to include the indirect impact in the second year of the pandemic) and the two post-pandemic periods (to assess the indirect impact at one and two years after the pandemic). The effect sizes were calculated as mean difference (MD) or odds ratio (OR) with 95% cluster-robust confidence interval (CI). A sensitivity analysis investigated the influence of age and sex on the outcome measures by adding the interaction terms period*age and period*sex in the regression models described above (Supplementary Material). Statistical significance was set at 5%. The statistical analysis was carried out using R 4.5 (R Foundation for Statistical Computing, Vienna, Austria) [6].

### 2.6. Ethics

The study adhered to Resolution no. 9/2016 of the Italian Data Protection Authority (Authorisation no. 9/2016), which permits personal data processing for scientific research. This regulation allows the use of health-related data in aggregate form for medical, biomedical, and epidemiological studies. It also states that obtaining informed consent is not required if contacting data subjects to provide information is impossible due to organizational constraints, especially considering the time elapsed since the data were initially collected. All analyses were performed on aggregated and anonymized data.

## 3. Results

### 3.1. Patients

The analysis included 4938 diagnoses of cutaneous melanoma in the study periods. Overall, the study sample included 2763 men and 2175 women aged 18–98 years (mean 60.6 years, SD 15.2). Clinical-pathological characteristics at different time points are summarized in Table 1.

### 3.2. Outcome Measures

**Figure 2:**
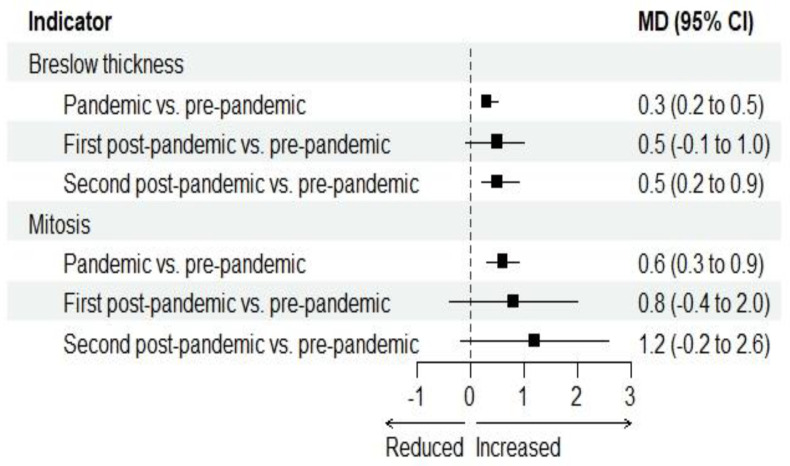
Breslow thickness and mitosis in the pandemic period (March 2021–February 2022), the first post-pandemic period (March 2022–February 2023) and the second post-pandemic period (March 2023–February 2024) compared to the pre-pandemic period (March 2019–February 2020): forest plots. CI: confidence interval. MD: mean difference.

**Figure 3:**
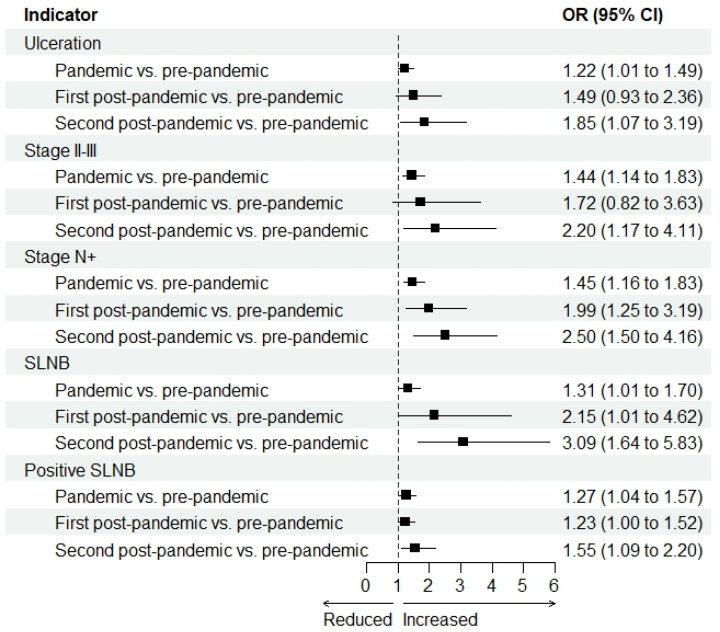
Ulceration, tumor stage, sentinel lymph node biopsy (SLNB) and positive SLNB in the pandemic period (March 2021–February 2022), the first post-pandemic period (March 2022–February 2023) and the second post-pandemic period (March 2023–February 2024) compared to the pre-pandemic period (March 2019–February 2020): forest plots. CI: confidence interval. OR: odds ratio.

Breslow thickness increased from the pre-pandemic period to the pandemic period (MD 0.3 mm, 95% CI 0.2 to 0.5; *p* < 0.0001) and the second post-pandemic period (MD 0.5 mm, 95% CI 0.2 to 0.9; *p* = 0.001) (Figure 2 and Supplementary Table S1). Mitosis increased from the pre-pandemic period to the pandemic period (MD 0.6 unit/mm^2^, 95% CI 0.3 to 0.9; *p* = 0.0001), but the change was not statistically significant (Figure 2 and Supplementary Table S1). Moreover, the presence of ulceration increased from the pre-pandemic period to the pandemic period (OR 1.22, 95% CI 1.01 to 1.49; *p* = 0.04) and the second post-pandemic period (OR 1.85, 95% CI 1.07 to 3.19; *p* = 0.03) (Figure 3 and Supplementary Table S1). Diagnoses of stage II–III increased from the pre-pandemic period to the pandemic period (OR 1.44, 95% CI 1.13 to 1.83; *p* = 0.003) and the second post-pandemic period (OR 2.20, 95% CI 1.17 to 4.11; *p* = 0.01) (Figure 3 and Supplementary Table S1). Also, N+ stage diagnoses increased from the pre-pandemic period to the pandemic period (OR 1.45, 95% CI 1.16 to 1.83; *p* = 0.001), the first post-pandemic period (OR 1.99, 95% CI 1.25 to 3.19; *p* = 0.003), and the second post-pandemic period (OR 2.50, 95% CI 1.50 to 4.16; *p* = 0.0004) (Figure 3 and Supplementary Table S1). Finally, the proportion of patients who underwent SLNB increased from the pre-pandemic period to the pandemic period (OR 1.31, 95% CI 1.01 to 1.70; *p* = 0.04), the first post-pandemic period (OR 2.15, 95% CI 1.01 to 4.62; *p* = 0.04), and the second post-pandemic period (OR 3.09, 95% CI 1.64 to 5.83; *p* = 0.0005) (Figure 2 and Supplementary Table S1). The proportion of patients with positive SLNB among those who underwent SLNB increased from the pre-pandemic period to the pandemic period (OR 1.27, 95% CI 1.01 to 1.57; *p* = 0.02) and the second post-pandemic period (OR 1.55, 95% CI 1.09 to 2.20; *p* = 0.01) (Figure 3 and Supplementary Table S1).

### 3.3. Age- and Sex-Adjusted Sensitivity Analyses

The sensitivity analyses showed that age and sex did not influence how the outcome measures changed during the study periods, apart from age and positive SLNB (Supplementary Material). In fact, older age was associated with decreased odds of positive SLNB in the first post-pandemic year (OR 0.99, 95% cluster-robust CI 0.98 to 0.99; *p* = 0.006), but not in the pandemic period (*p* = 0.11) or the second post-pandemic year (*p* = 0.10).

## 4. Discussion

The results of this large, Italian, multicenter, retrospective study indicate that the clinical presentation of primary cutaneous melanoma remained more advanced overall not only during the pandemic period but also during the first and second post-pandemic periods, compared with the pre-pandemic baseline. Breslow thickness, ulceration, higher tumor stage (II–III), and the proportion of patients undergoing SLNB and testing positive all showed a persistent worsening trend that did not return to pre-pandemic levels even four years after the onset of COVID-19. These results extend the previous observation that melanoma diagnoses were more advanced during the first pandemic year, likely due to delays in patients presenting for evaluation of concerning skin lesions. These findings suggest a persistent shift in melanoma presentation over time, which may be associated with pandemic-related disruptions as well as broader healthcare system and epidemiological factors. Importantly, given the retrospective and observational design of this study, these findings should be interpreted as associations rather than causal relationships. In the post-pandemic period, a progressively systemic bottleneck emerged, substantially constraining access to both primary and specialist care. During and after the pandemic, many primary care centers adopted appointment-only systems, reducing walk-in access to general practitioners. A study found that some changes intended to improve access, in fact, and impair it, requiring patients to align with and have the capabilities needed to use such systems. Some changes in how appointments are booked require people to have specific resources to use them (such as a telephone with credit, the ability to phone at the time called, or language capabilities to discuss needs) [7]. In fact, although these measures were necessary for infection control, they could limit or discourage the spontaneous access to primary care for evaluation [8], especially for those deemed low priority. Patients with suspicious lesions are often perceived by patients as low-priority concerns, largely because they are typically asymptomatic, may exhibit subtle or gradual changes, and are not perceived as immediately threatening overall health. Consequently, individuals may defer seeking medical evaluation [9].

Furthermore, even after restrictions were relaxed, ongoing issues such as cumulative delays, lengthy waiting lists, backlogs, and triage protocols continued to hinder prompt access to healthcare. One study, in fact, measures the extent to which waiting times and volume changed over time before and after COVID-19 in OECD countries (between 2016–2023), and found that relative to 2019, the waiting time to treatment increased on average by 5.9%, 8.6%, 14.7% and 12.9% in the four years following COVID-19 [10].

These challenges extended to dermatologist appointment diagnostic timelines and contributed to the observed rise in tumor thickness and cases presenting at more advanced stages.

Moreover, in Italy, we observe the “great escape” of professionals from the National Health Service, which often offers remuneration inferior to that in private practice [11] and COVID-19 further exacerbated these shortages by causing burnout and prompting retirements, resulting in a progressive reduction in the number of dermatologists, plastic surgeons, and surgical oncologists. Consequently, limited specialist availability led to prolonged waiting times for both diagnostic dermatology services and surgical management. These constraints may have contributed to the higher proportions of ulcerated tumors, increased mitotic activity, and higher rates of nodal metastasis observed in the later post-pandemic years.

Moreover, a significant shift in the types of patients presenting to melanoma centers appears to have occurred. During the pandemic and in the subsequent years, symptomatic or rapidly evolving lesions were more likely to prompt medical attention, while routine skin checks for asymptomatic individuals or patients under surveillance for prior melanoma were frequently postponed. This shift represents a major limitation of the study, as it may have led to an overrepresentation of biologically aggressive tumors and an underrepresentation of early-stage lesions, potentially contributing to an overestimation of the observed differences [12].

The persistence of more advanced melanoma at diagnosis cannot be attributed solely to pandemic-related health system disruptions; it also intersects with broader epidemiological trends that have been emerging in recent decades. Melanoma incidence has been increasing steadily across Europe and globally, particularly among younger and middle-aged adults. This upward trend, driven partly by increased UV exposure and improved diagnostic awareness, has resulted in a growing pool of individuals at risk, thereby amplifying the consequences of any increase in waiting times for visits. The pandemic, occurring against the backdrop of this epidemiological rise, may have amplified these effects. In fact, a Greek study evaluating superficial spreading melanoma thickness found a significant 2% per year increase (percent of change: 2.0, 95% CI: 0.2, 3.7) from 2010 to 2021, while a similar trend in nodular melanoma (NM) thickness was observed [13]. This could also be due to long-term shifts in sun exposure habits—including increased outdoor activities, intermittent intense UV exposure, and artificial tanning practices—that have contributed to a higher baseline risk of melanoma over recent decades [14]. During the pandemic, particularly during lockdown transitions, changes in outdoor behavior patterns (e.g., increased outdoor recreation when restrictions allowed) may have influenced UV exposure and accelerated the progression of pre-existing lesions that subsequently presented at more advanced stages. Moreover, Italy’s aging population contributes to a higher burden of melanoma, with older individuals exhibiting both higher incidence and thicker lesions contributing to more advanced presentations [15].

Overall, these findings align with modeling studies that predicted the COVID-19 pandemic would lead to delayed melanoma diagnoses, more advanced disease at presentation, and worse long-term outcomes [1]. The higher stage at diagnosis carries prognostic implications and may translate into increased mortality rates in the years ahead if this trend is not reversed through public health awareness efforts.

### Strengths and Limitations

The multicenter design and the large sample size enhance the generalizability across Italian melanoma referral centers. However, the retrospective design precludes assessment of patient-level behavioral factors, socioeconomic barriers, and timelines of symptom development. Nonetheless, the consistency of findings across diverse institutions underscores the systemic and multifactorial nature of the phenomenon. Further longitudinal research is recommended to validate these findings and to incorporate an extended follow-up duration. Additionally, the study design does not allow for adjustment of all potential confounders, including healthcare access, patient behavior, and socioeconomic factors, which may have influenced the observed trends.

## 5. Conclusions

This study provides evidence of a persistent shift toward more advanced melanoma at diagnosis in Italy in the years following the COVID-19 pandemic. These patterns may reflect not only healthcare disruptions but also broader epidemiological trends, including rising melanoma incidence, demographic changes, and evolving UV exposure behaviors.

Addressing this sustained shift toward late diagnosis will require reinforced public health campaigns, improved access to dermatologic care, and strategic actions to counteract specialist shortages and restore systematic early detection pathways.

## Figures and Tables

**Table 1:** Summary of the outcome measures in the study periods.

Outcome Measure	Pre-Pandemic Period (n = 1637)	Pandemic Period (n = 1290)	First Post-Pandemic Period (n = 1096)	Second Post-Pandemic Period (n = 915)
Breslow thickness, mm: mean (SD)	1.6 (2.7)	1.9 (2.7)	2.1 (2.9)	2.1 (3.3)
Mitosis, unit/mm^2^: mean (SD)	2.2 (4.4)	2.8 (4.8)	3.0 (5.3)	3.4 (6.0)
Tumor stage II–III: n/N (%)	321/1576 (20.4%)	337/1254 (26.9%)	335/1096 (30.6%)	329/915 (36.0%)
Stage N+: n/N (%)	118/1590 (7.4%)	132/1260 (10.5%)	151/1094 (13.8%)	153/915 (16.7%)
Ulceration: n/N (%)	234/1437 (16.3%)	213/1108 (19.2%)	211/941 (22.4%)	212/801 (26.5%)
Patients who underwent SLNB: n/N (%)	591/1591 (37.1%)	548/1256 (43.6%)	546/975 (56.0%)	530/820 (64.6%)
Patients with positive SLNB: n/N (%)	115/588 (19.5%)	129/545 (23.7%)	126/546 (23.1%)	145/530 (27.4%)

SD: standard deviation. SLNB: sentinel lymph node biopsy. Pre-pandemic period: from March 2019 to February 2020; pandemic period: from March 2021 to February 2022. First post-pandemic period (first year): from March 2022 to February 2023. Second post-pandemic period (second year): from March 2023 to February 2024.

## Data Availability

The datasets presented in this study can be found in online repositories. The names of the repository/repositories and accession number(s) can be found here: https://zenodo.org/records/19384746 (accessed on 2 April 2026).

## Supplementary Materials

The following supporting information can be downloaded at: https://www.mdpi.com/article/10.3390/jcm15072715/s1, Supplementary Table S1. Results from the regression models.
