# Supplementary material for: Clinical Characteristics at the Diagnosis of New Primary Melanoma in Italy: A Multicenter Retrospective Study Before and After the COVID-19 Pandemic
Source: J Clin Med. 2026 Apr 3;15(7):2715. doi: 10.3390/jcm15072715 (PMC13073478; doi:10.3390/jcm15072715)
Supplement: Supplementary file 1 [file jcm-15-02715-s001.zip › jcm-4178963-supplementary.pdf]

SUPPLEMENTARY MATERIAL

Numerical results from the regression models in the main analysis

Supplementary Table S1. Results from the regression models: the effect sizes estimated the changes from the pre-pandemic to the pandemic period, from the pre-pandemic to the post-pandemic period (1st year) and from the pre-pandemic to the post-pandemic period (2nd year)

Outcome measure	Effect size	Pandemic vs. pre-pandemic	Post-pandemic (1st year) vs. pre-pandemic	Post-pandemic (2nd year) vs. pre-pandemic
Breslow thickness, mm	MD (95% cluster-robust CI)	0.3 (0.2 to 0.5) *	0.5 (-0.1 to 1.0)	0.5 (0.2 to 0.9) *
Mitosis, unit/mm^2	MD (95% cluster-robust CI)	0.6 (0.3 to 0.9) *	0.8 (-0.4 to 2.0)	1.2 (-0.2 to 2.6)
Tumor stage II-III	OR (95% cluster-robust CI)	1.44 (1.13 to 1.83) *	1.72 (0.82 to 3.63)	2.20 (1.17 to 4.11) *
Tumor stage N+	OR (95% cluster-robust CI)	1.45 (1.16 to 1.83) *	1.99 (1.25 to 3.19) *	2.50 (1.50 to 4.16) *
Ulceration	OR (95% cluster-robust CI)	1.22 (1.01 to 1.49) *	1.49 (0.93 to 2.36)	1.85 (1.07 to 3.19) *
Patients who underwent SLNB: n/N (%)	OR (95% cluster-robust CI)	1.31 (1.01 to 1.70) *	2.15 (1.01 to 4.62) *	3.09 (1.64 to 5.83) *
Patients with positive SLNB: n/N (%)	OR (95% cluster-robust CI)	1.27 (1.04 to 1.57) *	1.23 (1.00 to 1.52)	1.55 (1.09 to 2.20) *

CI: confidence interval. MD: mean difference. SLNB: sentinel lymph node biopsy. OR: odds ratio. *p<0.05. Pre-pandemic period: from March 2019 to February 2020; pandemic period: from March 2021 to February 2022. First post-pandemic period (first year): from March 2022 to February 2023. Second post-pandemic period (second year): from March 2023 to February 2024.

Age-adjusted sensitivity analysis

Age did not influence the changes of Breslow thickness from the pre-pandemic to the pandemic period ($p=0.41$), the first post-pandemic year ($p=0.84$) or the second post-pandemic year ($p=0.35$). However, older age was associated with higher Breslow thickness overall (MD 0.03 mm, 95% cluster-robust CI 0.02 to 0.04; $p<0.0001$).

Age did not influence the changes of mitosis from the pre-pandemic to the pandemic period ($p=0.81$), the first post-pandemic year ($p=0.75$) or the second post-pandemic year ($p=0.34$). However, older age was associated with higher mitosis overall (MD 0.05 unit/mm^2, 95% cluster-robust CI 0.03 to 0.06; $p<0.0001$).

Age did not influence the changes in the proportion of tumor stage II-III from the pre-pandemic to the pandemic period ($p=0.65$), the first post-pandemic year ($p=0.13$) or the second post-pandemic year ($p=0.32$). However, older age was associated with higher proportion of tumor stage II-III overall (OR 1.02, 95% cluster-robust CI 1.01 to 1.03; $p<0.0001$).

Age did not influence the changes in the proportion of tumor stage N+ from the pre-pandemic to the pandemic period ($p=0.20$), the first post-pandemic year ($p=0.38$) or the second post-pandemic year ($p=0.49$). Moreover, age was not associated with tumor stage N+ overall ($p=0.25$).

Age did not influence the changes in the proportion of ulceration from the pre-pandemic to the pandemic period ($p=0.16$), the first post-pandemic year ($p=0.32$) or the second post-pandemic year ($p=0.45$). However, older age was associated with higher proportion of ulceration overall (OR 1.03, 95% cluster-robust CI 1.02 to 1.03; $p<0.0001$).

Age did not influence the changes in the proportion of SLNB from the pre-pandemic to the pandemic period ($p=0.48$), the first post-pandemic year ($p=0.80$) or the second post-pandemic year ($p=0.32$). Moreover, age was not associated with SLNB overall ($p=0.81$).

Age influenced the changes in the proportion of positive SLNB during the study periods. In fact, older age was associated with decreased odds of positive SLNB in the first post-pandemic year (OR 0.99, 95% cluster-robust CI 0.98 to 0.99; $p=0.006$), but not in the pandemic period ($p=0.11$) or the second post-pandemic year ($p=0.10$).

Sex-adjusted sensitivity analysis

Sex did not influence the changes of Breslow thickness from the pre-pandemic to the pandemic period (p=0.49), the first post-pandemic year (p=0.84) or the second post-pandemic year (p=0.92). However, males were associated with higher Breslow thickness overall (MD 0.3 mm, 95% cluster-robust CI 0.1 to 0.4; p=0.007).

Sex did not influence the changes of mitosis from the pre-pandemic to the pandemic period (p=0.91), the first post-pandemic year (p=0.56) or the second post-pandemic year (p=0.79). However, male sex was close to be associated with higher mitosis overall (MD 0.3 unit/mm^2, 95% cluster-robust CI 0.0 to 0.6; p=0.05).

Sex did not influence the changes in the proportion of tumor stage II-III from the pre-pandemic to the pandemic period (p=0.35), the first post-pandemic year (p=0.53) or the second post-pandemic year (p=0.59). However, males were associated with higher proportion of tumor stage II-III overall (OR 1.19, 95% cluster-robust CI 1.03 to 1.37; p=0.01).

Sex did not influence the changes in the proportion of tumor stage N+ from the pre-pandemic to the pandemic period (p=0.60), the first post-pandemic year (p=0.81) or the second post-pandemic year (p=0.64). Moreover, sex was not associated with tumor stage N+ overall (p=0.16).

Sex did not influence the changes in the proportion of ulceration from the pre-pandemic to the pandemic period (p=0.98), the first post-pandemic year (p=0.95) or the second post-pandemic year (p=0.56). However, males were associated with higher proportion of ulceration overall (OR 1.35, 95% cluster-robust CI 1.17 to 1.55; p<0.0001).

Sex did not influence the changes in the proportion of SLNB from the pre-pandemic to the pandemic period (p=0.92), the first post-pandemic year (p=0.53) or the second post-pandemic year (p=0.10). However, male sex was close to be associated with SLNB overall (OR 1.13, 95% cluster-robust CI 0.99 to 1.29; p=0.07).

Sex did not influence the changes in the proportion of positive SLNB from the pre-pandemic to the pandemic period (p=0.55), the first post-pandemic year (p=0.52) or the second post-pandemic year (p=0.88). Moreover, sex was not associated with positive SLNB overall (p=0.15).
